# The Validity of a Smartphone-Based Application for Assessing Cognitive Function in the Elderly

**DOI:** 10.3390/diagnostics15010092

**Published:** 2025-01-03

**Authors:** Jin-Young Min, Duri Kim, Hana Jang, Hyunjoo Kim, Soojin Kim, Seungbo Lee, Yae-eun Seo, Ye-jin Kim, Jong-yoon Kim, Kyoung-Bok Min

**Affiliations:** 1 Veterans Medical Research Institute, Veterans Health Service Medical Center, Seoul 05368, Republic of Korea; minymink@naver.com (J.-Y.M.); fbw9c3@naver.com (D.K.); hanaj.nurse@gmail.com (H.J.); ahyunjookim@gmail.com (H.K.); wls5580@naver.com (S.K.); 2 Beluga Corp, Chang-up-ro, 54, Seongnam-si 13449, Gyeonggi-do, Republic of Korea; jake.lee@belugatech.io (S.L.); dodlsdl24@gmail.com (Y.-e.S.); isla@belugatech.io (Y.-j.K.); hank.kim@belugatech.io (J.-y.K.); 3 Department of Preventive Medicine, College of Medicine, Seoul National University, 103 Daehak-ro, Jongno-gu, Seoul 03080, Republic of Korea

**Keywords:** cognitive assessment, digitalized tool, validation, feasibility, cognitive impairment

## Abstract

**Background/Objectives:** The early detection of individuals at risk of cognitive impairment is a clinical imperative. With the recent advancement of digital devices, smartphone application-based cognitive assessment is considered a promising tool for cognitive screening and monitoring inside and outside the clinic. This study examined whether a smartphone-based cognitive assessment, Brain OK, was valid for evaluating cognitive performance and identifying people at risk of cognitive impairment. **Methods:** We recruited 88 study participants aged over 60. They completed two cognitive tests with the Montreal Cognitive Assessment (MoCA), a validated paper-and-pencil cognitive screening tool, and Brain OK, a smartphone-based cognitive testing application. To examine convergent validity, we conducted analyses of Spearman correlations between MoCA and BrainOK, a Bland–Atman plot with regression analysis, and the area under the curve (AUC). **Results:** There was a significant positive association between Brain OK and the MoCA total score, with a coefficient of 0.9044 (SE = 0.057, t = 15.750, *p* < 0.001). The Bland–Altman plot represented a reasonable level of agreement between the two tests. We conducted the AUC analysis of Brain OK to compare the cognitively normal and impaired groups. The AUC value for the Brain OK score of 13.5 was the highest at 0.941. The sensitivity and specificity were 0.958 and 0.925, respectively. **Conclusions:** The smartphone app-based Brain OK test was feasible for assessing cognitive function and acceptable for identifying subjects with cognitive impairment. The results suggest Brain OK complements traditional in-person cognitive assessments and may help enhance cognitive health dialogue between doctors and patients.

## 1. Introduction

The global human population is aging rapidly. The population share of people aged 65 years and older is expected to increase from 761 million in 2021 to 1.6 billion by 2050 [1]. Consequently, the proportion of people aged 80 years or older is also on the rise.

Age is the most significant risk factor for cognitive impairment, and the prevalence of some forms of cognitive impairment has also increased [2]. Cognitive impairment is the most common health issue among the elderly, with a global prevalence ranging from 5.1% to 41% across different nations [2]. Cognitive impairment, which refers to mild cognitive impairment (MCI) and dementia, interferes with an individual’s independent everyday activities and imposes economic and social burdens [3]. Several pharmacological treatments are available to prevent or delay cognitive impairment, especially Alzheimer’s dementia (AD); however, their benefits are usually modest [4]. Timely diagnosis is essential to optimize patient care, practical support, and treatment [2,3,5]. However, evidence shows that most patients with AD or MCI are unfortunately diagnosed when the disease has already progressed to the moderate or severe stage. Thus, developing tools and methods to facilitate the early detection of cognitive impairment risk is critical.

Many clinical cognitive assessment methods such as cerebrospinal fluid or neuroimaging biomarkers are invasive, time-consuming, and expensive [6], hindering early screening in primary care or community settings. In contrast, paper-and-pencil cognitive assessment is a relatively low-cost and non-invasive method for identifying cognitive diseases and impairments [7]. Brief cognitive testing, such as the Montreal Cognitive Assessment (MoCA) and Mini-Mental State Examination (MMSE), has traditionally been considered a time-efficient strategy for evaluating comprehensive cognitive abilities [7]. Although these brief tests have low sensitivity for diagnosing patients with AD or MCI, they are still essential for assessing people who require cognitive evaluation for a final diagnosis and appropriate treatment and care [8].

The need for the early detection of cognitive impairment and the advancement of digital technologies have introduced cognitive testing methods using smartphone devices, which are becoming ubiquitous [8,9]. Smartphone-based cognitive assessment allows older adults to be more engaged in cognitive screening and monitoring both inside and outside the clinic [9]. The methods provide advantages over traditional cognitive testing such as cost and time savings, more detailed response metric recordings, and automated data collection and scoring. There is limited evidence proving the feasibility and validity of smartphone-based cognitive assessments in older adults without dementia or with some form of dementia [8,10,11]. To effectively monitor cognitive function or detect early cognitive decline, it is essential to conduct comprehensive assessments that encompass a wide range of brain functional domains. However, previous studies have primarily focused on evaluating cognitive function based on specific domains (e.g., memory) or a limited set of test items while assessing the feasibility and validity of smartphone-based cognitive assessments. Given that smartphone-based assessments are increasingly being used to complement traditional paper-and-pencil cognitive tests, their ability to distinguish between cognitively healthy individuals and those with cognitive impairment is a critical consideration. Nevertheless, most related studies to date have reported results based on specific patient populations (e.g., individuals with multiple sclerosis or schizophrenia) rather than the general population. This limitation may hinder the broader application of cognitive screening services to more diverse groups. Consequently, research on the effectiveness of smartphone-based cognitive assessments as an alternative to traditional paper-and-pencil tests remains scarce and inconclusive.

Brain OK (Beluga Inc., Gyeonggi-do, Republic of Korea) is a novel smartphone-based cognitive assessment application. It was designed to evaluate the comprehensive cognitive function of older adults in terms of their overall and specific cognitive abilities, including attention, memory, visuospatial ability, language, and executive function. Each test item in Brain OK is based on a validated paper-and-pencil cognitive task [12]. The Brain OK prototype was developed by a collaborating interdisciplinary team consisting of professionals from the fields of medicine, computer science, psychology, neuroscience, and public health.

The current study aimed to examine whether Brain OK, a smartphone-based cognitive assessment tool, is valid for evaluating cognitive performance and identifying individuals at risk of cognitive impairment. We investigated the congruence of cognitive performance measured between Brain OK and MoCA as the validated paper-and-pencil cognitive screening tool. We also measured the sensitivity and specificity of Brain OK against MoCA to discriminate between cognitively impaired individuals and cognitively normal controls.

## 2. Materials and Methods

### 2.1. Study Population

We recruited 88 study participants aged over 60 years who visited the Seoul Veterans Health Service Medical Center in the Republic of Korea between July and October 2024. The inclusion criteria were older adults aged 60 years and above (1) who voluntarily agreed to participate in the current study with an understanding of the study aim and procedures, and (2) who had no difficulties in writing and reading with sufficient vision and hearing. The exclusion criteria were as follows: (1) those who were diagnosed with neurodegenerative diseases (e.g., Alzheimer’s, Parkinson’s, and dementia); (2) those who had impaired decisional capacity due to cognitive and medical problems; (3) those who had severe physical and psychiatric diseases (e.g., cancer, brain infarction, cerebral hemorrhage) that could significantly impede research; and (4) those who did not complete the cognitive testing measured by paper-and-pencil-based and smartphone app-based assessments. Cognitive function assessments were conducted with the MoCA test administered first, followed by the Brain OK test. A minimum 30 min break was provided between the two tests to minimize potential learning effects and ensure that the first test did not influence the results of the second.

The study protocol was reviewed and approved by the Institutional Review Board of the Veterans Health Service Medical Center (BOHUN IRB 2024-06-013). Informed consent was obtained from all participants before beginning the research procedures.

### 2.2. Paper- and Pencil-Cognitive Assessment: Montreal Cognitive Assessment (MoCA)

The MoCA is a brief cognitive screening instrument with high sensitivity and specificity for detecting MCI and the early signs of dementia [13]. It is a paper-and-pencil tool comprising 11 subset tasks assessing seven cognitive domains: executive/visuospatial function, naming, attention, language, abstraction, recall, and orientation. The MoCA is administered for approximately 10 min, and its score is derived by adding points for each domain, with a maximum score of 30 points. The cutoff value for a normal MoCA score is 23, with scores of 23 and below indicating MCI. A trained professional administered and scored the MoCA in this study.

### 2.3. Smartphone App-Based Cognitive Assessment: Brain OK

Brain OK is a smartphone-based cognitive testing application designed for older adults to assess cognitive function using their own smartphones. The assessment software was developed for use on touchscreen smartphones and is compatible with Apple iPhones and Android phones. It comprises 11 tests covering five cognitive domains: attention, memory, visuospatial ability, language, and executive function. Brain OK consists of a practice phase and a main phase and takes 20 min to administer.

All participants performed the Brain OK test in a quiet, relatively distraction-free environment. During the practice phase, the participants received a brief explanation of the test, followed by practice trials. They could choose to repeat the practice. Otherwise, they proceeded to the main phase. Supplementary Table S1 presents a comprehensive overview of the assessment, including illustrative examples of the visual displays utilized in Brain OK. Additionally, Supplementary Table S2 highlights the advantages of Brain OK in comparison to existing smartphone-based cognitive tests. Brain OK offers thorough neurocognitive evaluations spanning all five cognitive domains, with a strong emphasis on accessibility, efficiency, and adaptability. By providing clearly defined cutoff scores, Brain OK supports the reliable detection of MCI and is tailored to accommodate individuals with diverse educational backgrounds and varying levels of smartphone proficiency.

### 2.4. Statistical Analysis

The sample size for evaluating the statistical difference between the cognitive tests used in clinical settings and Brain OK was determined using the G*Power program. With a power of 90%, α = 0.05, and an effect size of 0.3, a total of 88 participants was calculated.

To assess the convergent validity between Brain OK and the MoCA, all 88 participants completed both tests, and their measurement values were subsequently compared. Spearman correlation and Bland–Altman analyses were performed to evaluate the degree of agreement between Brain OK and MoCA. Spearman correlations were conducted between the total Brain OK and MoCA total scores. A Bland–Altman plot with regression analysis is a method of data plotting used to compare differences between mean values and estimate an agreement limit within a 95% CI. This suggests a possible relationship between the measurements and the true value (i.e., proportional bias), wherein if a proportional bias (*p* < 0.05) exists, the two measurements do not agree equally through the range of the measurements (i.e., the limits of agreement will depend on the actual measurement). We also calculated the area under the curve (AUC), a summary metric of the receiver operating characteristic (ROC) curve reflecting Brain OK’s ability to distinguish between the MCI group and normal controls. The MCI group was defined as individuals with MoCA scores < 23. In diagnostic value studies, 0.9 ≤ AUC is interpreted as very good performance, while AUC values below 0.80, even if statistically significant, are interpreted as indicating a limited clinical usability of the test. All statistical analyses were performed using R 4.4.1 (R Foundation for Statistical Computing, Vienna, Austria) and Python 3.8 (Python Software Foundation). Statistical significance was set at *p* ≤ 0.05.

## 3. Results

Table 1 shows the demographic characteristics of the study population according to the presence of cognitive impairment. Except for sex, there were significant differences in age, years of education, and MoCA scores between cognitively normal and cognitively impaired individuals.

Table 2 shows the success ratios of each Brain OK subtest for the normal control and MCI groups. Overall, a significant difference in the accuracy rate was observed between them, indicating that the accuracy rate was higher in the cognitively normal group than in the MCI group.

Table 3 shows the Spearman correlation between the Brain OK subtest and MoCA total score. Overall, the correlation between each subtest of the Brain OK and MoCA total scores for all subjects was significant. A positive correlation was found for all subjects in the bubble cancellation, block counting, naming, and comprehension quizzes (r = 0.307, *p* = 0.004; r = 0.414, *p* < 0.001; r = 0.347, *p* = 0.001; and r = 0.312, *p* = 0.003). A significant correlation was found for all subjects in Password memorization I, II, Right–left orientation test, Drum game I, II, Memorization of sentence—recall, and the Subtraction train (r = 0.577, *p* < 0.001; r = 0.536, *p* < 0.001; r = 0.524, *p* = 0.004; r = 0.632, *p* < 0.001; r = 0.566, *p* < 0.001; r = 0.593, *p* < 0.001; and r = 0.596, *p* < 0.001).

**Figure 1:**
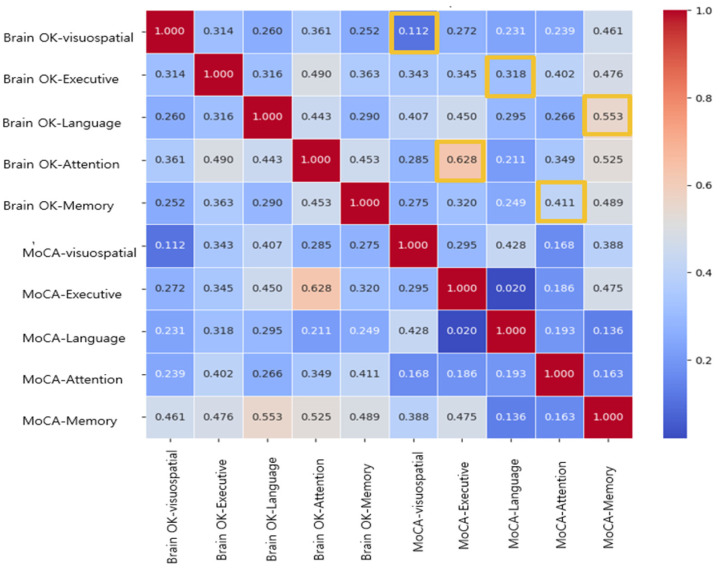
Correlation heatmap of subdomains between Brain OK and MoCA.

Figure 1 presents a correlation heatmap illustrating the associations between the Brain OK and MoCA subdomains. Stronger correlations are observed in domains such as attention and language, particularly between Brain OK_Attention and MoCA_Executive function (r = 0.628) and between Brain OK_Language and MOCA_Memory (r = 0.553). There was moderate correlation between Brain OK_Executive and MOCA_Attention (r = 0.318). In contrast, weaker correlations are noted for visuospatial and executive function parameters, such as Brain OK_Visuospatial and MOCA_Visuospatial (r = 0.112) and Brain OK_Executive and MOCA_Language (r = 0.318).

**Figure 2:**
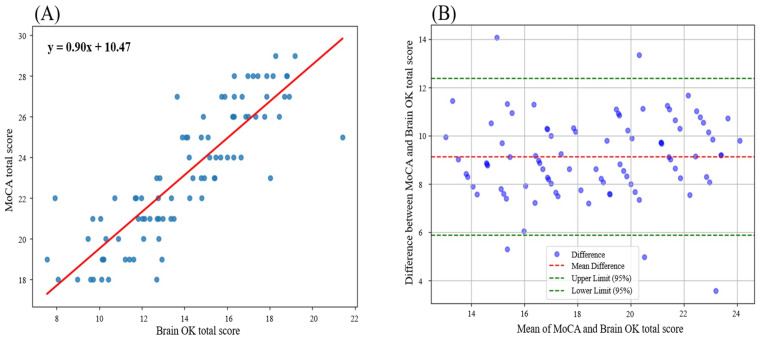
Correlation scatter plot (**A**) and Bland–Altman plot (**B**) for Brain OK vs. MoCA. Each blue dot represents an individual data point, reflecting observed values for the two variables. The red line indicates the linear regression line, demonstrating the overall trend between the two scores.

Figure 2A shows the correlation scatter plot with a linear regression line. Regression analysis showed a significant positive association between Brain OK and the MoCA total score, with a coefficient of 0.9044 (SE = 0.057, t = 15.750, *p* < 0.001). An R-squared value was 0.743, indicating that the model exhibited a strong explanatory power. In addition, the overall model was statistically significant (F = 248.1, *p* < 0.001), indicating a good fit for the data.

The Bland–Altman plot (Figure 2B) shows a mean difference of 9.122 between MoCA and Brain OK, indicating a systematic bias between the two methods. However, this bias does not necessarily diminish the validity of Brain OK as a cognitive assessment tool. The consistent deviation may reflect inherent differences in the scoring systems or test design of the two measures. Importantly, 94.318% of data points fall within the 95% confidence limits [5.873, 12.370], suggesting a reasonable level of agreement. This high percentage of agreement indicates that, despite the systematic bias, Brain OK demonstrates reliable performance relative to MoCA, supporting its potential utility for cognitive screening.

**Figure 3:**
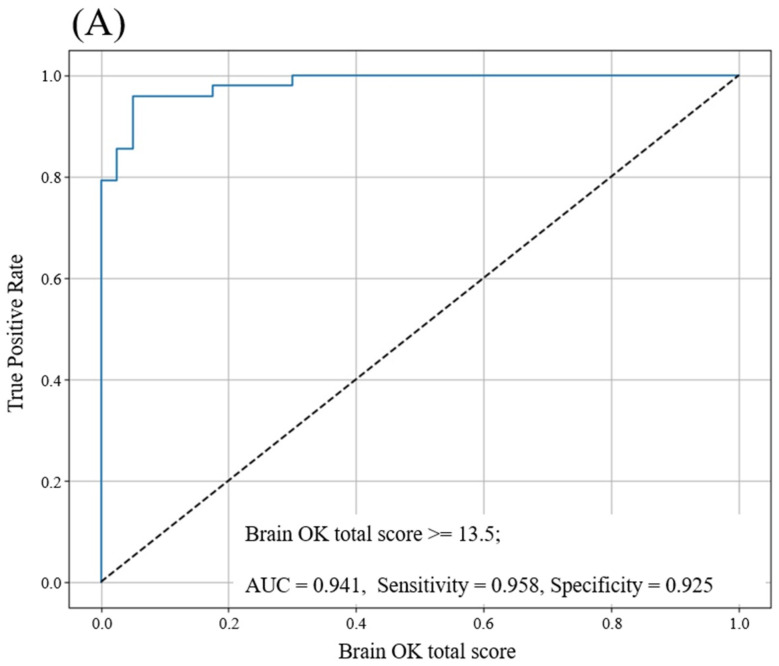

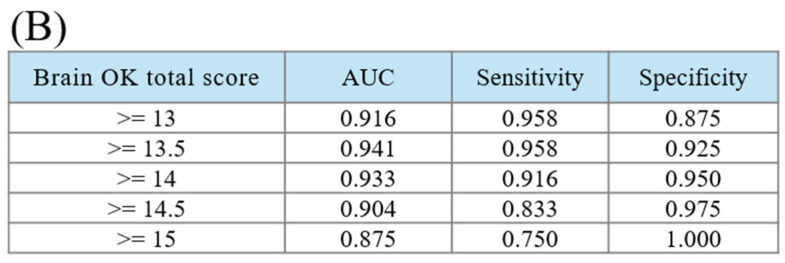
The ROC curve for the Brain OK total score in the comparison between the normal control and MCI groups (**A**) and description (**B**).

Based on the MoCA score, the 88 subjects were classified as either MCI (*n* = 39) or cognitively normal (*n* = 49) people. We performed an ROC analysis of Brain OK to compare the ability to differentiate cognitively normal and cognitively impaired individuals. An ROC curve was plotted (Figure 3A) to determine the optimal cutoff value. As shown in Figure 3B, the AUC values for the Brain OK scores of 13, 13.5, 14, 14.5, and 15 were 0.916, 0.941, 0.933, 0.904, and 0.875, respectively. The highest AUC value was observed at a score of 13.5. The sensitivity and specificity were 0.958 and 0.925, respectively, for a score of 13.5, indicating a high level of accuracy in distinguishing between cognitively normal and MCI groups.

## 4. Discussion

Our study shows that Brain OK, a smartphone-based application, is feasible for assessing cognitive function and acceptable for identifying subjects with cognitive impairment. The domain-specific and domain-general cognitive performance assessment developed in Brain OK was significantly correlated with the MoCA and total scores, underlining its reliability as a cognitive assessment tool and validating the use of smartphone-based cognitive evaluations. With a cutoff score of 13.5 out of 22 on Brain OK, it exhibited outstanding performance, achieving an AUC value of 0.94 in discriminating between the normal control and MCI groups. This demonstrates Brain OK’s potential as an effective screening tool for the early detection of cognitive decline. Its accessibility and scalability make it particularly valuable in resource-limited or time-constrained environments, in which traditional cognitive assessments may be less feasible.

Despite the advantages of smartphone-based cognitive assessments, such as being rapid, cost-effective, and sensitive measures for detecting dementia-related cognitive impairment in clinical and research settings [9], few studies have validated smartphone app-based cognitive assessments [8,14,15]. Brouillette et al. (2013) investigated the validity of a smartphone-based application, the Color–Shape Test (CST), for the assessment of cognitive function in 57 non-demented elderly individuals. In the sample, CST scores were significantly associated with MMSE (r = 0.515, *p* < 0.0001) and the cognitive processing speed tests Digit Span (r = 0.427, *p* < 0.0001), Trail Making Test (r = −0.651, *p* < 0.00001), and Digit Symbol Test (r = 0.508, *p* < 0.0001) [14]. Jongstra et al. (2017) developed the smartphone-based app iVitality, designed to evaluate five digital versions of cognitive tests (Memory-Word, Trail Making, Stroop, Reaction Time, and Letter-N-Back). The authors investigated the feasibility and validity of iVitality in elderly individuals at an increased risk of dementia during a 6-month follow-up period. A moderated correlation (r = 0.36~0.62, *p* < 0.001) between the smartphone-based test and the conventional test was found in Stroop and Trail Making tests [15]. Öhman et al. (2022) evaluated the validity of two mobile app-based memory tasks, the Mnemonic Discrimination Task for Objects and Scenes (MDT-OS) and the long-term delayed Object-In-Room Recall Task (ORR-LDR), by comparing them with conventional cognitive tests in 172 non-demented older adults [10]. There was a significant correlation between two mobile app-based memory tasks and the conventional cognitive test scores (r  =  0.32~0.44, *p*  <  0.001). Recently, De Anda-Duran et al. (2024) compared the congruence between a smartphone-based assessment (DANA, Defense Automated Neurocognitive Assessment) and traditional neuropsychological tests in two diverse cohorts [8]. The DANA includes three tasks: the Code Substitution, the Go/No-Go, and the Simple Reaction Time tests. The authors found that the global cognitive Z-score measured using traditional neuropsychological tests was significantly associated with DANA tasks, and the observed results were consistent in two different cohorts. In our analysis, individual cognitive tests within Brain OK demonstrated weak-to-moderate correlations with the MoCA, ranging from 0.307 to 0.632, with relatively stronger correlations observed in executive function tasks (e.g., Drum Game I and Drum Game II). Notably, the total Brain OK score exhibited a high correlation of 0.9044 with the MoCA, reflecting strong agreement with this widely used conventional cognitive assessment. Although direct comparisons with previous studies are challenging due to differences in methodologies and resources, the correlation of 0.9044 represents a higher level of concordance, highlighting the robustness of Brain OK as a reliable cognitive assessment tool. Unlike prior studies, which often focus on isolated domains such as memory or processing speed, Brain OK demonstrated relatively consistent correlations across multiple cognitive domains. This indicates its versatility and potential as a comprehensive, multi-domain tool for cognitive evaluation.

Another concern is the potential of smartphone-based cognitive tests to identify individuals with impaired cognition. Several studies using smartphone devices for cognitive assessment have focused on detecting and monitoring cognitive dysfunction in specific disorders [16,17]. For example, Maillart et al. (2020) [16] and Lam et al. (2022) [17] evaluated the validity of a smartphone-adapted Symbol Digit Modalities Test (sSDMT) in patients with multiple sclerosis (MS). When the authors combined a composite smartphone assessment of information processing speed with walking, manual dexterity, and low-contrast visual acuity, the AUC value to distinguish healthy control and MS patients was 0.92 [16]. Subsequent studies [17] found a moderate ability with an AUC value of 0.713 when only the sSDMT was used. In a study on identifying subjects with schizophrenia versus healthy controls, a gamified mobile phone version of the Trails-B cognitive assessment showed high performance, with an AUC of 0.94 [18]. Staffaroni et al. (2024) measured smartphone application (app)-based executive functioning and associative memory tasks for frontotemporal lobar degeneration (FTLD) evaluations [19]. They found that smartphone tests accurately differentiated individuals with dementia from controls (AUC = 0.93; 95% CI, 0.90–0.96) and were more sensitive to early symptoms (AUC = 0.82; 95% CI, 0.76–0.88) than the MoCA (AUC = 0.68; 95% CI, 0.59–0.78). Smartphone-based cognitive assessments have demonstrated varying levels of validity across different cognitive domains and target populations. Our study adds to this growing body of evidence by validating Brain OK, a multi-domain assessment tool, with a high AUC value of 0.941 (95% CI: 0.892–0.991), a sensitivity of 0.958, and a specificity of 0.925. These results indicate that Brain OK provides robust performance in detecting cognitive impairment, outperforming many existing smartphone-based tools, such as the sSDMT (AUC = 0.713) and MoCA (AUC = 0.68 in FTLD evaluations). The relatively high correlation between Brain OK and MoCA, along with its superior AUC value, may be attributed to its comprehensive approach to assessing multiple cognitive domains, including executive function and processing speed. This aligns with the findings of Staffaroni et al. (2024) [19], who emphasized the importance of multi-domain assessments in improving the sensitivity of smartphone-based tools for the early detection of cognitive impairment. Despite these promising findings, this study is limited by its reliance on MoCA cutoff scores (<23) to define cognitive impairment, which may introduce bias. Although a systematic review and meta-analysis demonstrated that a MoCA cutoff score of 23 lowers the false-positive rate and shows overall good diagnostic accuracy in identifying MCI in general practice [20], it is necessary to confirm the reproducibility of our results based on accurate clinical diagnosis and an evaluation of the cognitive impairment group.

This study has several important limitations. First, the small sample size, recruitment from a single center, and the restricted age range of participants (62–88 years) with cognitive concerns. This may have introduced selection bias, potentially limiting the generalizability of the findings. Furthermore, the inclusion of participants who expressed interest in smartphone-based cognitive tests may have resulted in self-selection bias, as the sample likely overrepresented individuals predisposed to using such technology. Second, before the smartphone-based assessments, the participants received short face-to-face instructions for the tests to limit the influence of technical issues. Face-to-face learning may have affected participants’ motivation and performance. Third, the tests were conducted only once. The strength of mobile cognitive testing is its ability to examine day-to-day variability in cognitive performance and its relationship to real-world outcomes or cognitive decline. Therefore, repeated cognitive measurements may reduce the misclassification of the current cognitive function status and represent the true extent of functional decline. Finally, barriers to smartphone use often exist among older adults, and smartphone-based tests may be affected by their ability to use smartphones (i.e., touchscreen manipulation and search for health information). However, we did not collect information on smartphone usage-related ability, so we do not know whether they have difficulty using smartphones, which hinders inspection, or vice versa. These limitations should be addressed in future studies.

In conclusion, our study demonstrated that the smartphone app-based Brain OK test is both feasible and valid. Brain OK effectively differentiated between individuals with and without cognitive impairment, suggesting its potential as a scalable, accessible, and efficient solution for cognitive screening, particularly in resource-limited settings. Unlike traditional paper-and-pencil tests, Brain OK leverages smartphone technology to bridge gaps in cognitive health monitoring by enabling early detection and follow-up assessments, especially in underrepresented or remote populations. Future studies should focus on validating Brain OK’s effectiveness in predicting cognitive decline over time and exploring its integration with broader health monitoring systems for a more holistic approach to patient care.

## Figures and Tables

**Table 1:** Demographic characteristics of study participants.

Variables	All Population (*n* = 88)	Normal Controls (*n* = 48)	MCI Group (*n* = 40)	*p*-Value *
Female, *n* (%)	58 (65)	33 (37.5)	25 (28.4)	0.696
Age (year), mean (SD)	74.42 (5.32)	72.62 (5.00)	76.58 (4.95)	<0.0001
Years of education, mean (SD)	10.96 (3.92)	13.15 (3.33)	8.35 (2.83)	<0.0001
MoCA scores, mean (SD)	23.19 (3.23)	25.71 (1.84)	20.18 (1.45)	<0.0001

* *p*-values were calculated using a Student’s *t*-test for continuous variables and the chi-square test for categorical variables to compare the two groups: normal cognition and the MCI group (MoCA scores < 23).

**Table 2:** The mean success ratios in the Brain OK test for the normal control and MCI groups.

Domain	Test	All Population	Normal Cognition	MCI Group	*p*-Value *
Attention	Bubble cancellation	0.90 (0.09)	0.93 (0.06)	0.86 (0.10)	<0.001
	Password memorization I	0.28 (0.21)	0.37 (0.23)	0.16 (0.09)	<0.001
	Password memorization II	0.19 (0.14)	0.25 (0.14)	0.11 (0.09)	<0.001
	Subtraction Train	0.65 (0.30)	0.83 (0.22)	0.44 (0.23)	<0.001
Visuospatial function	Block counting	0.63 (0.27)	0.73 (0.27)	0.51 (0.23)	<0.001
Executive function	Drum game I	0.77 (0.26)	0.92 (0.07)	0.58 (0.29)	<0.001
	Drum game II	0.82 (0.24)	0.95 (0.09)	0.67 (0.29)	<0.001
Memory	Memorization of sentence—recall	0.23 (0.27)	0.36 (0.30)	0.08 (0.11)	<0.001
Language	Right–left orientation	0.55 (0.33)	0.69 (0.28)	0.38 (0.31)	<0.001
	Naming Test	0.63 (0.21)	0.68 (0.19)	0.56 (0.22)	0.009
	Comprehension quiz	0.90 (0.11)	0.93 (0.10)	0.85 (0.12)	0.002

* *p*-values were calculated using a Student’s *t*-test to compare the two groups: normal cognition and the MCI group (MoCA scores < 23).

**Table 3:** Spearman’s correlation between Brain OK and MoCA total scores.

Domain	Test	Correlation Coefficients	*p*-Value
Attention	Bubble cancellation	0.307	0.004
	Password memorization I	0.547	<0.001
	Password memorization II	0.536	<0.001
	Subtraction train	0.596	<0.001
Visuospatial Function	Block counting	0.414	<0.001
Executive Function	Drum game I	0.632	<0.001
	Drum game II	0.566	<0.001
Memory	Memorization of sentence—recall	0.593	<0.001
Language	Right–left orientation	0.524	0.004
	Naming test	0.347	0.001
	Comprehension quiz	0.312	0.003

## Data Availability

The datasets analyzed during the current study are available from the corresponding author upon reasonable request.

## Supplementary Materials

The following supporting information can be downloaded at: https://www.mdpi.com/article/10.3390/diagnostics15010092/s1, Table S1. A detailed description of each test and screenshots of the Brain OK. Table S2. A detailed description of each test and screenshots of the Brain OK. References [8,10,14,21,22,23,24,25,26,27,28,29,30,31,32,33] are cited in the supplementary materials.
