# Supplementary material for: The Validity of a Smartphone-Based Application for Assessing Cognitive Function in the Elderly
Source: Diagnostics (Basel). 2025 Jan 3;15(1):92. doi: 10.3390/diagnostics15010092 (PMC11719899; doi:10.3390/diagnostics15010092)
Supplement: Supplementary file 1 [file diagnostics-15-00092-s001.zip › diagnostics-3359754-supplementary.pdf]

Supplement Table S1. Detailed description of each test and screenshots of the Brain OK

Domain	Subtest	Description	Reference
Attention	Bubble cancellation	•The bubbles, each containing one of several images, filled the screen while moving slowly upward. Participants were instructed to tap the bubbles that contained target images. Across two trials, a total of 49 bubbles were presented, of which 20 contained target images.<br>•The success ratio was calculated by dividing the number of successfully tapped target bubbles (‘S’) by the total number of attempts.<br>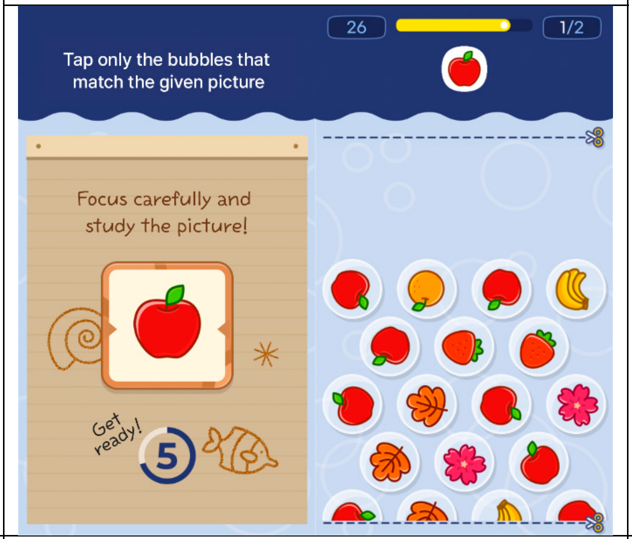	*Letter cancellation*<br><br>Della Sala et al., 1992 [21]
Attention	Password memorizatio n I	•Participants completed a digit span memory task, where they listened to a series of digits, memorized them, and entered them using an on-screen keypad. The task began with 3-digit sequences in stage 1, and the sequence length increased by one digit at each subsequent stage, reaching 9 digits by stage 7.<br>•Each stage was assigned a score ranging from 1 to 7 points. Participants were allowed one retry per stage upon failure; success was recorded whether achieved on the first attempt or the retry.<br><br>•The success ratio was calculated as the sum of scores for successful stages (‘S’) divided by the total possible score of 28.	*Digit span test - forward*<br><br>Leung et al., 2011 [22]

		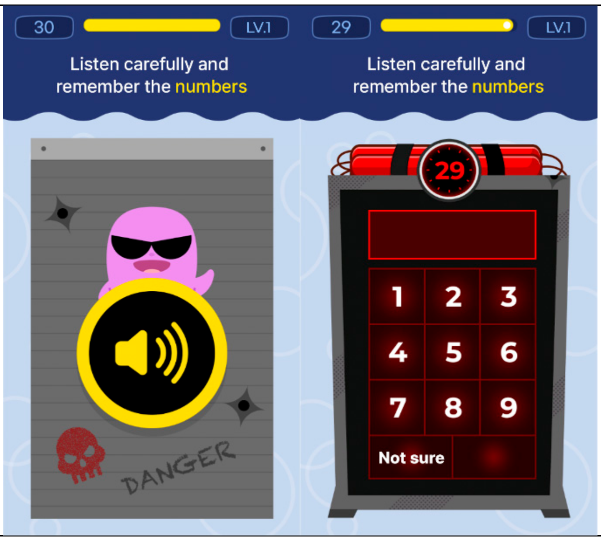 	
Attention	Password memorization II	•Similar to Password Memorization I, a series of digits were presented auditorily at each stage. However, in this test, participants were instructed to input the digits in reverse order. The sequence began with 2 digits in the first stage and increased incrementally to 8 digits by the 7th stage.<br><br>•Each stage was assigned a score ranging from 1 to 7 points. Participants were allowed one retry per stage upon failure; success was recorded whether achieved on the first attempt or the retry.<br><br>•The success ratio was calculated as the sum of scores for successful stages (‘S’) divided by the total possible score of 28.<br><br>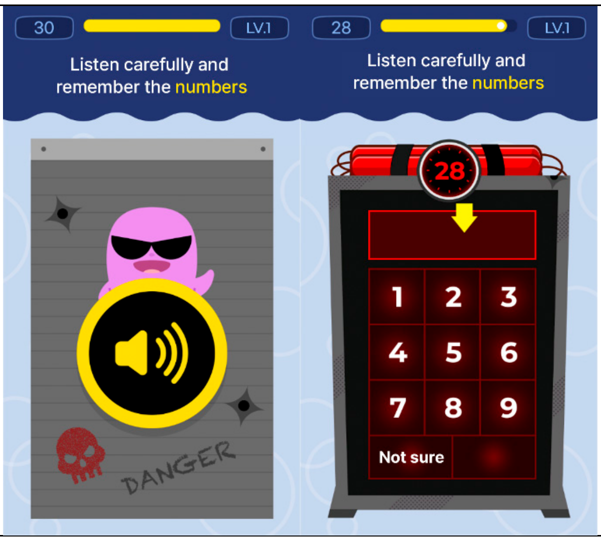 	*Digit span test – backward*<br><br>Leung et al., 2011 [22]
Attention	Subtraction train	•Participants continuously subtracted 7 from 100 a total of 5 times. Regardless of accuracy, the answer of the previous subtraction became the new starting point of the next formula.	*Serial seven*<br><br>Bristow et al., 2016 [23]

		•The success ratio was calculated as the number of correct answers out of a total of 5 attempts.<br>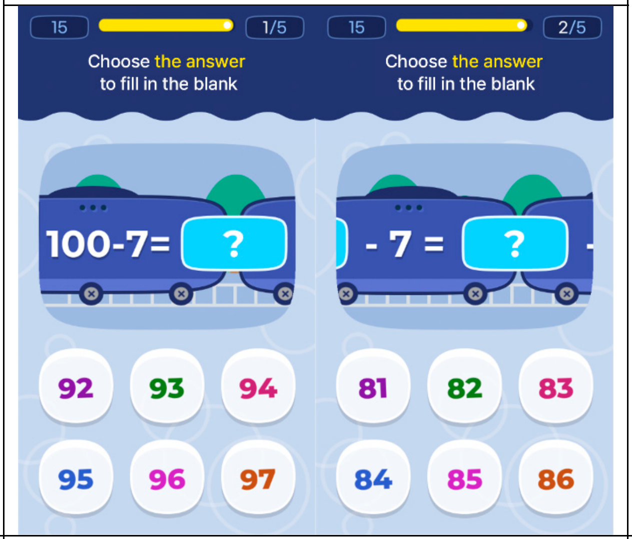	
Visuospatial	Block counting	•Participants were presented with a cube structure and tasked with counting the number of cubes. They selected the correct answer from five options or chose the "I don't know" button. The task consisted of 8 stages, with scores ranging from 1 to 8 points assigned progressively across the stages.<br>•Participants were allowed one retry per stage upon failure; success was recorded whether achieved on the first attempt or on the retry. However, failing twice in a single stage ended the task.<br>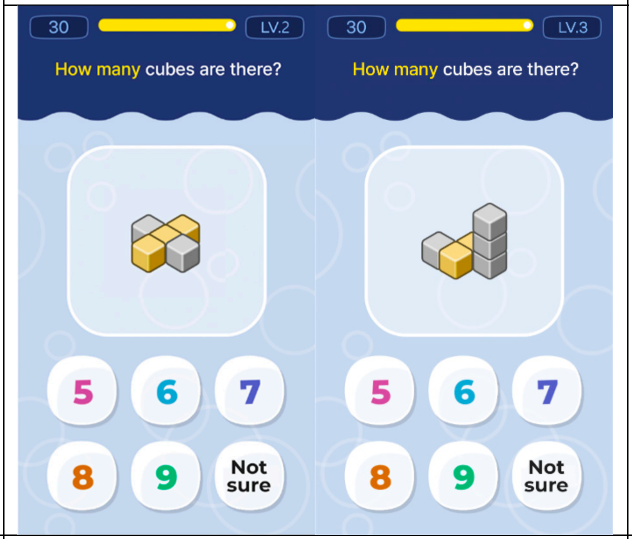	*VOSP - cube analysis*<br>Quental et al., 2013 [24]
Executive	Drum game I	•Participants tapped the drum using one or two fingers based on the number of spectators displayed in the seat. Specifically, they were instructed to use one finger if there were two spectators and two fingers if there was one	*Go/No-go test*<br>Wright et al., 2014 [25]

		spectator. A total of 20 trials were conducted, each with a three-second time limit.<br><br>•The success ratio was calculated as the number of correct answers out of a total of 20 attempts.	
		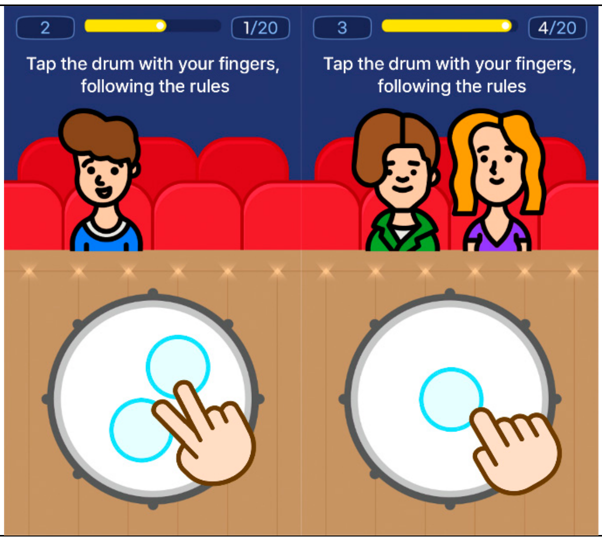	
Executive	Drum game II	•Similar to the Drum Game I, participants were instructed to touch the drum based on the number of spectators. However, in this version, they were specifically instructed not to touch the drum when two spectators were presented.<br><br>•The success ratio was calculated as the number of correct answers out of a total of 20 attempts.	*Go/No-go test*<br><br>Wright et al., 2014 [25]
Memory	Memorization of sentence - recall	•Participants repeated aloud the sentence they had memorized in the previous encoding and storage test. In the second trial, they were asked to recall four main keywords-name, residence, job, and work start time. If they failed in each keyword, four possible answers were presented and participants had to select the correct answer.<br><br>•The success ratio was calculated as the sum of correct answers divided by total score 8.	*K-CIST*<br><br>Ministry of Health and Welfare, 2021 [26];<br><br>Kintsch et al., 1990 [27]

		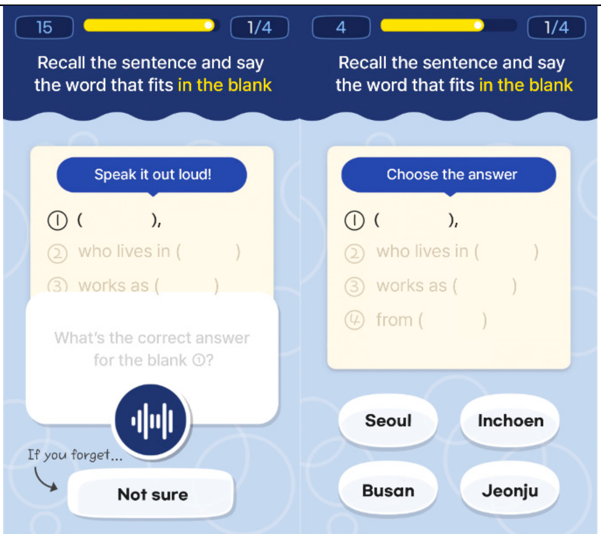	
Language	Right-left orientation	•Participants were presented with rabbits of different colors arranged around a round table. They were instructed to touch the rabbit positioned to the left or right of the pink rabbit. The rabbit locations were pseudorandomized.<br><br>•The number of rabbits increased across trials: 3 rabbits for the first three trials, 5 rabbits for the next two trials, and 6 rabbits for the final two trials. The task consisted of 7 trials, each with a binary outcome of success or failure.<br><br>•The success ratio was calculated as the number of successful trials divided by the total number of trials (7).<br><br>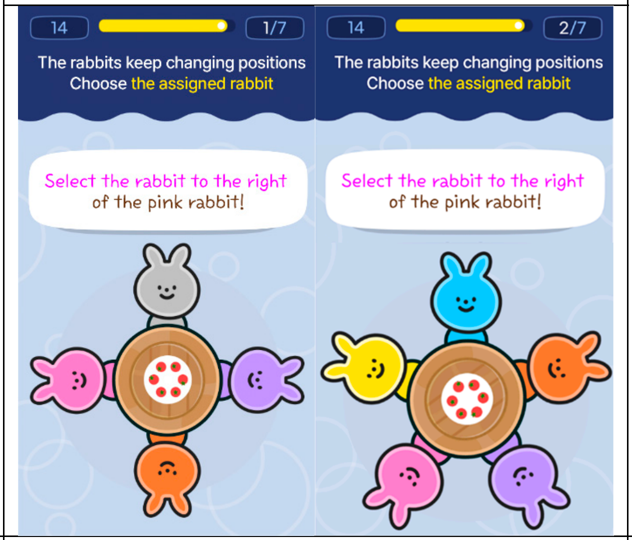	*Right-left orientation*<br><br>Ryu et al., 2023 [28]
Language	Naming test	•Participants were instructed to verbally name the presented image. They were informed that the first hint will be presented after 15 seconds and second hint after 22 seconds from image presentation. The first hint was a semantic hint, and the second hint	Boston naming test<br><br>Willers et al., 2008 [29]

		was the first letter of the answer. The presented images were those of a bed, an ostrich, and tweezers.<br><br>•The success ratio is calculated as the sum of correct answers divided by total score 6.<br><br>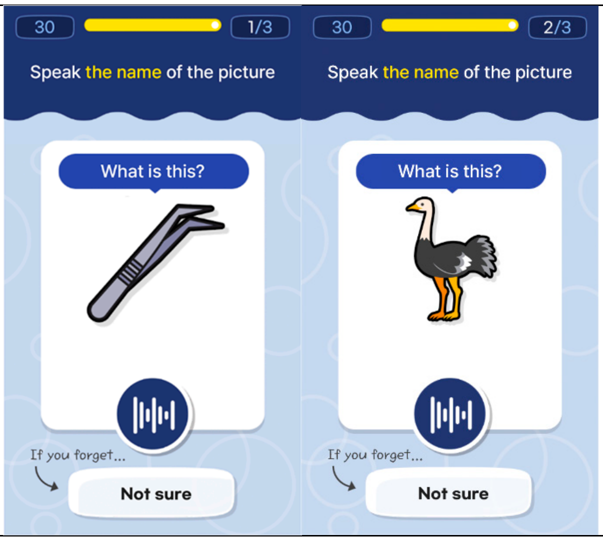 	
Language	Comprehensi on quiz	•Participants listened to sentences that were clearly identifiable as true or false. They were instructed to determine the accuracy of each sentence and select the 'Yes' button if it was true or the 'No' button if it was false. A total of 5 sentences were presented.<br>•The success ratio was calculated as the number of correct responses out of 5.<br><br>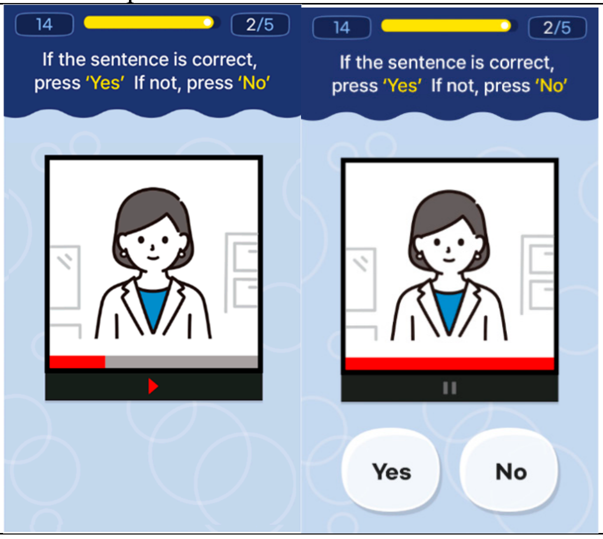 	Western aphasia battery - Yes/No Question<br><br>Kertesz et al., 2020 [30]

Supplement Table S2. Detailed description of each test and screenshots of the Brain OK

Existing assessment tool	Advantages of Brain OK
DANA: Defense Automated Neurocognitive Assessment [8]	•Assesses all five cognitive domains.<br>•Provides clear cutoff scores that distinguish between Mild Cognitive Impairment (MCI) and normal cognition.<br>•Demonstrates feasibility for individuals with lower educational levels and older adults with limited smartphone proficiency.
Cognitive Assessment for Dementia [31]	•Supports both AOS and iOS platforms, expanding accessibility beyond iPad devices.<br>•Identifies and distinguishes MCI accurately.
Smartphone-based color-shape test [14]	•Covers all five cognitive domains.<br>•Provides clear cutoff scores to support differentiation of cognitive impairments.
Computerized cognitive screening [32]	•Includes all five cognitive domains, with specific emphasis on language abilities.<br>•Provides clear cutoff scores for differentiation between MCI and normal cognition.<br>•Designed to be feasible for elderly individuals with lower educational backgrounds and limited smartphone experience.
Brain Health Assessment [33]	•Incorporates tasks that evaluate all five cognitive domains.<br>•Provides clear cutoff scores for cognitive impairment detection.
Mobile app-based memory tasks [10]	•Covers all five cognitive domains, including memory.<br>•Facilitates MCI detection within a shorter assessment time (~20 minutes).<br>•Adapts tasks with adjustable difficulty levels.<br>•Provides text and audio explanations for ease of understanding.
